# Supplementary figures and images for: Inhibition of Cpeb3 ribozyme elevates CPEB3 protein expression and polyadenylation of its target mRNAs and enhances object location memory
Source: eLife. 2024 Feb 6;13:e90116. doi: 10.7554/eLife.90116 (PMC10919898; doi:10.7554/eLife.90116)

**Figure 1**

**F**

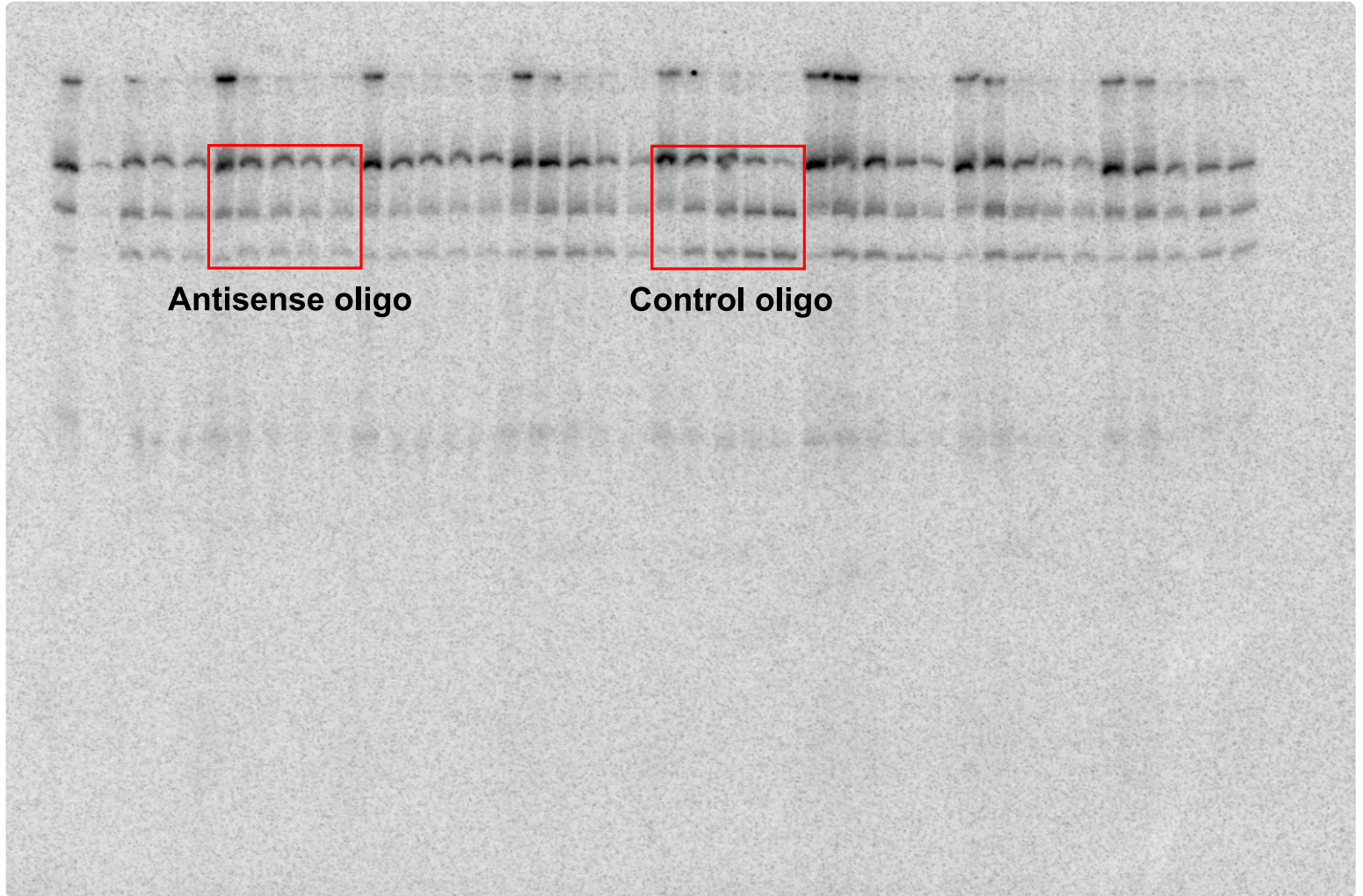

Supplement: Figure 1—source data 1. [file elife-90116-fig1-data1.zip › Fig. 1 source data/Fig. 1 source data-2.pdf]

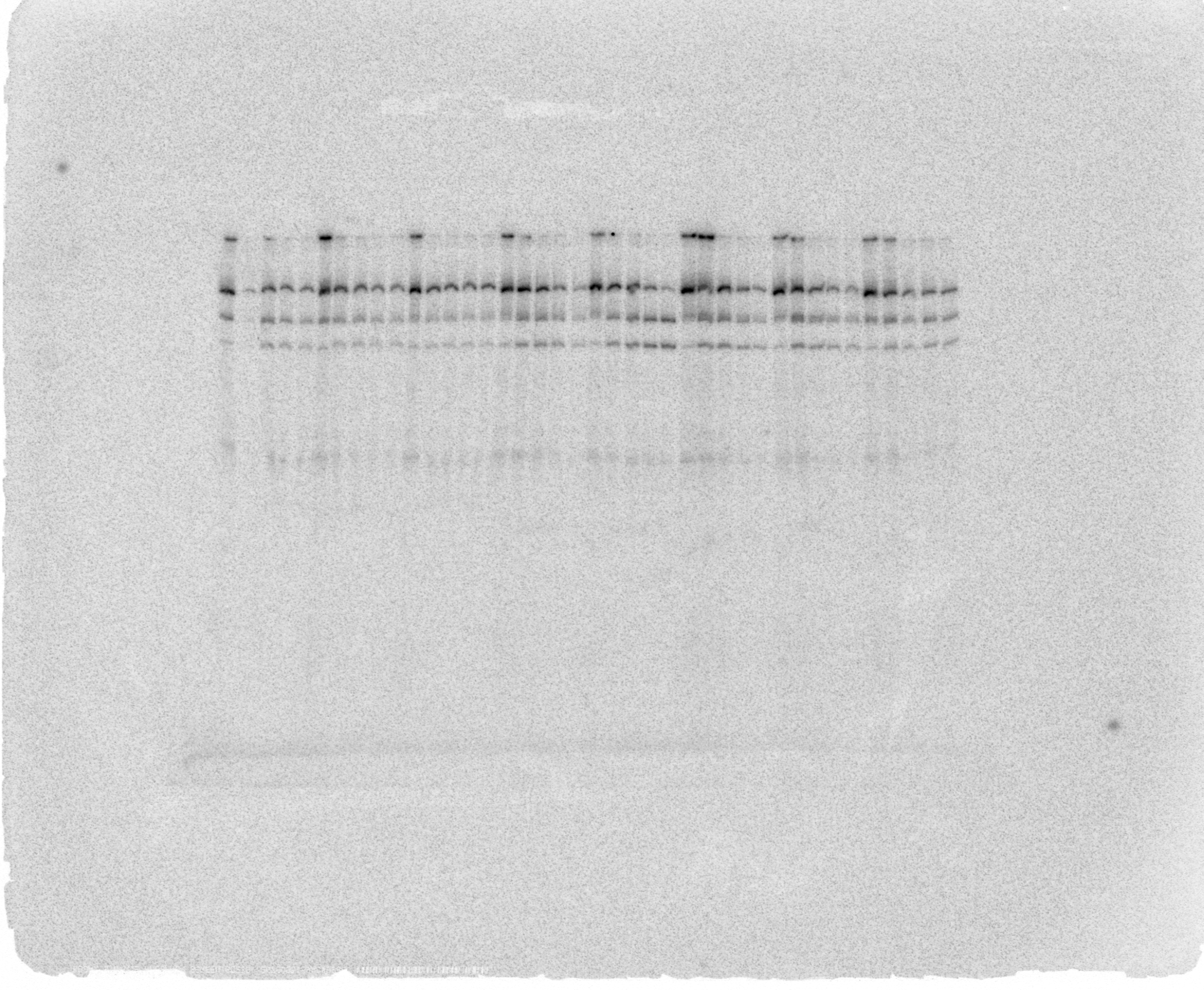

Supplement: Figure 1—source data 2. [file elife-90116-fig1-data2.zip › Fig.1 orignal image jpeg/20180413 - Kinetic Antisense tests.jpg]

**Figure 4**

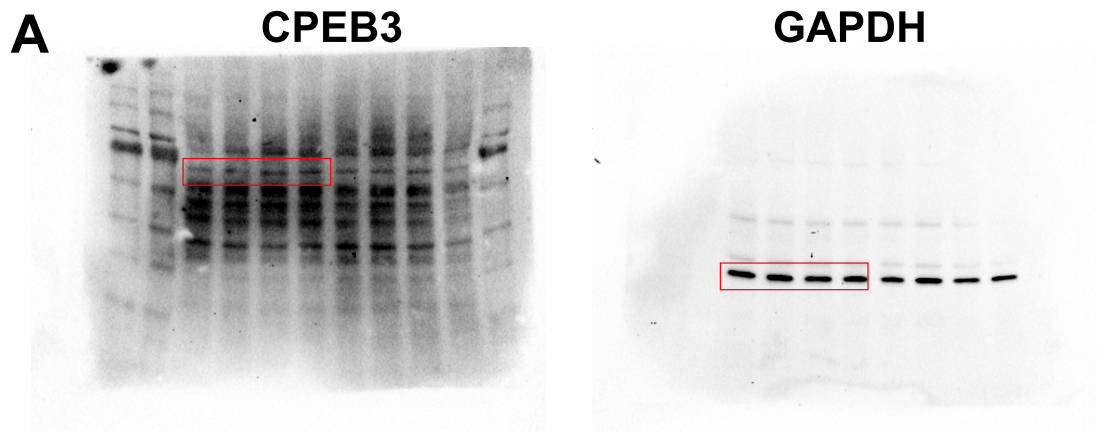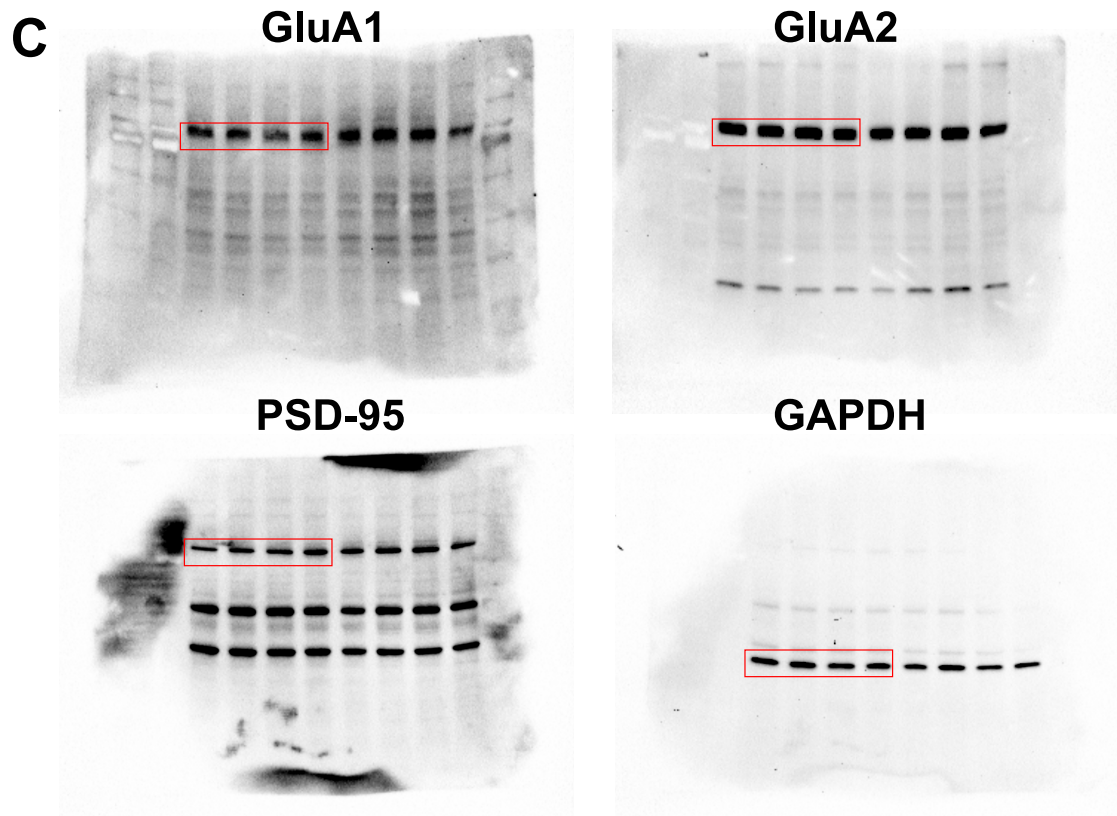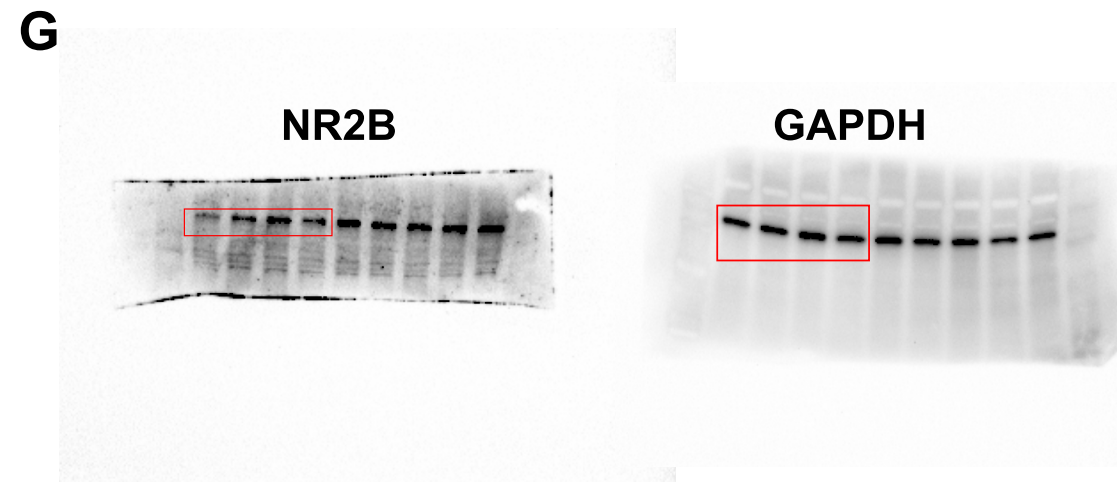

Supplement: Figure 4—source data 1. [file elife-90116-fig4-data1.zip › Fig. 4 source data/Fig.4 source data-2.pdf]

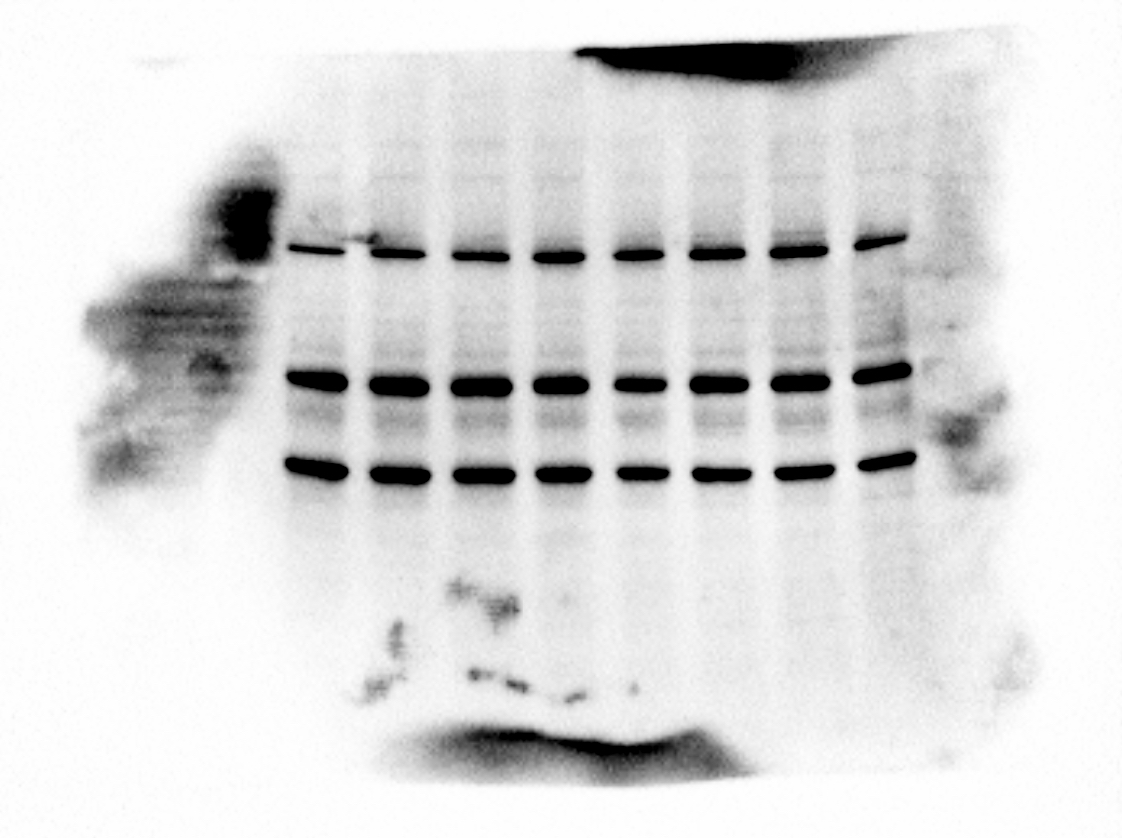

Supplement: Figure 4—source data 2. [file elife-90116-fig4-data2.zip › Fig.4 orignal images jpeg/2019-02-12 PSD95.jpg]

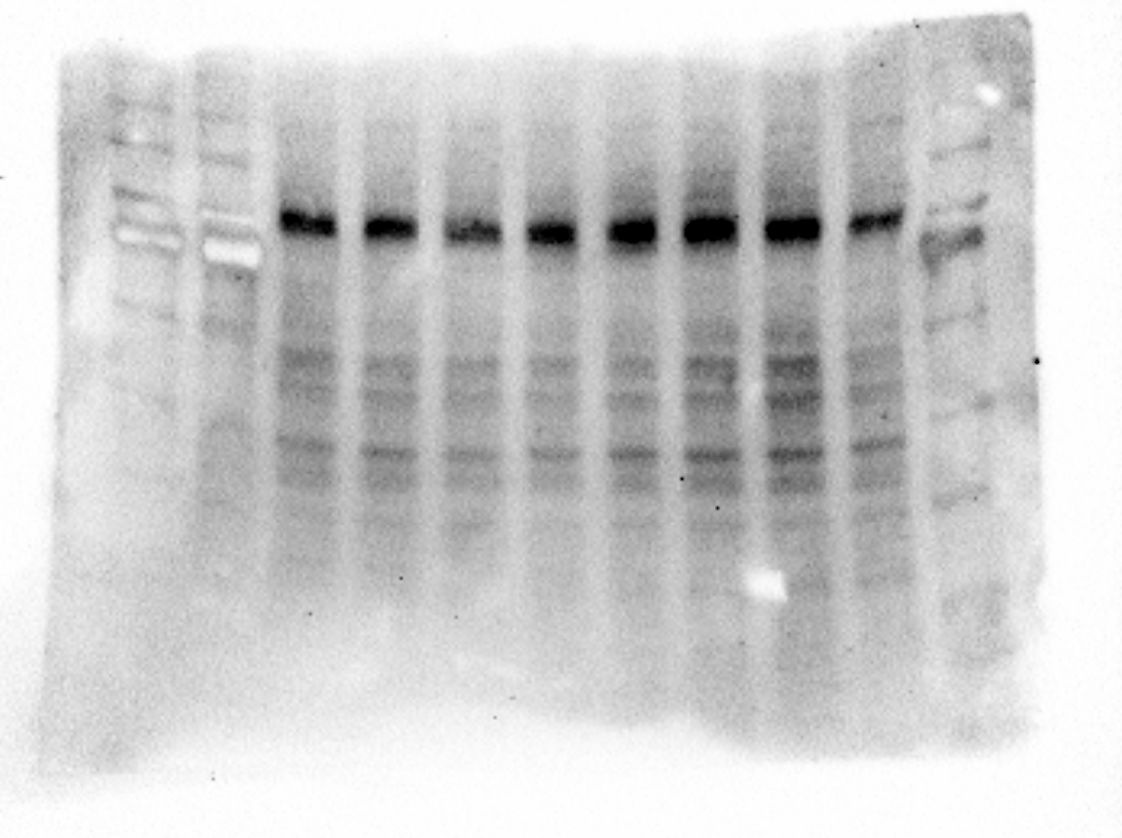

Supplement: Figure 4—source data 2. [file elife-90116-fig4-data2.zip › Fig.4 orignal images jpeg/2019-02-09 GluA1.jpg]

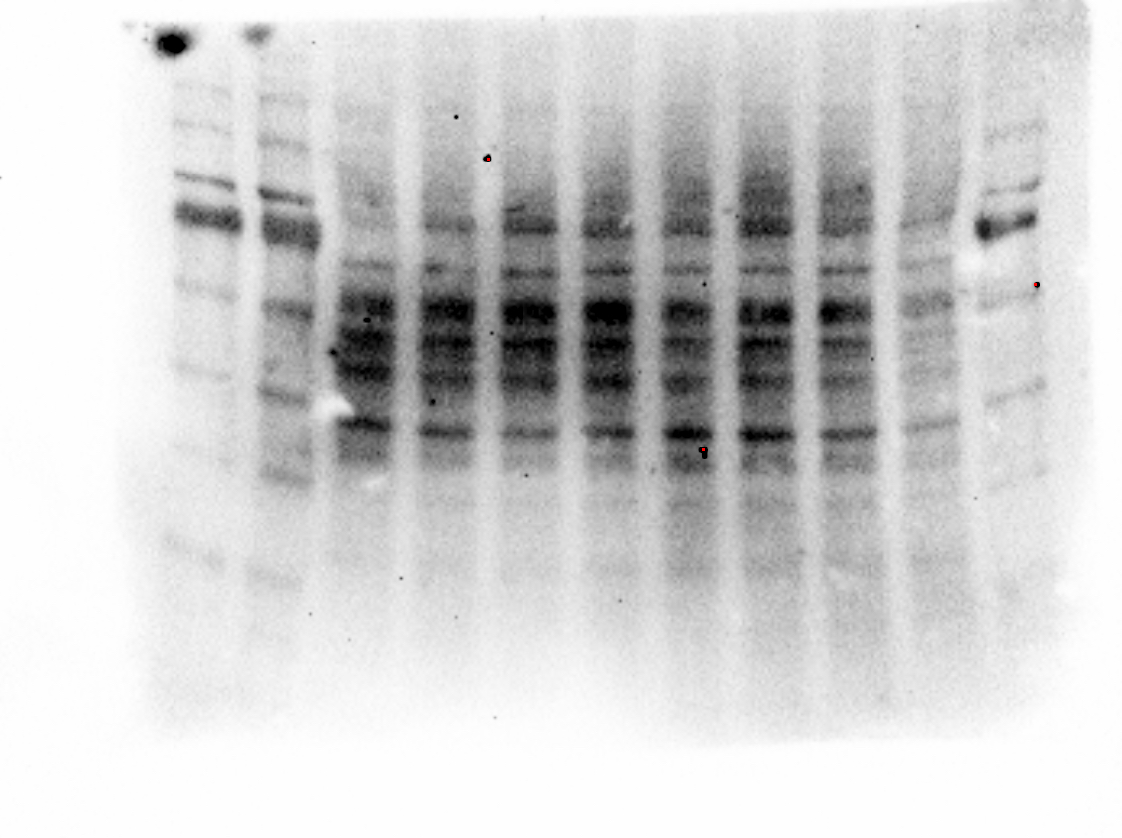

Supplement: Figure 4—source data 2. [file elife-90116-fig4-data2.zip › Fig.4 orignal images jpeg/2019-02-06_CPEB3.jpg]

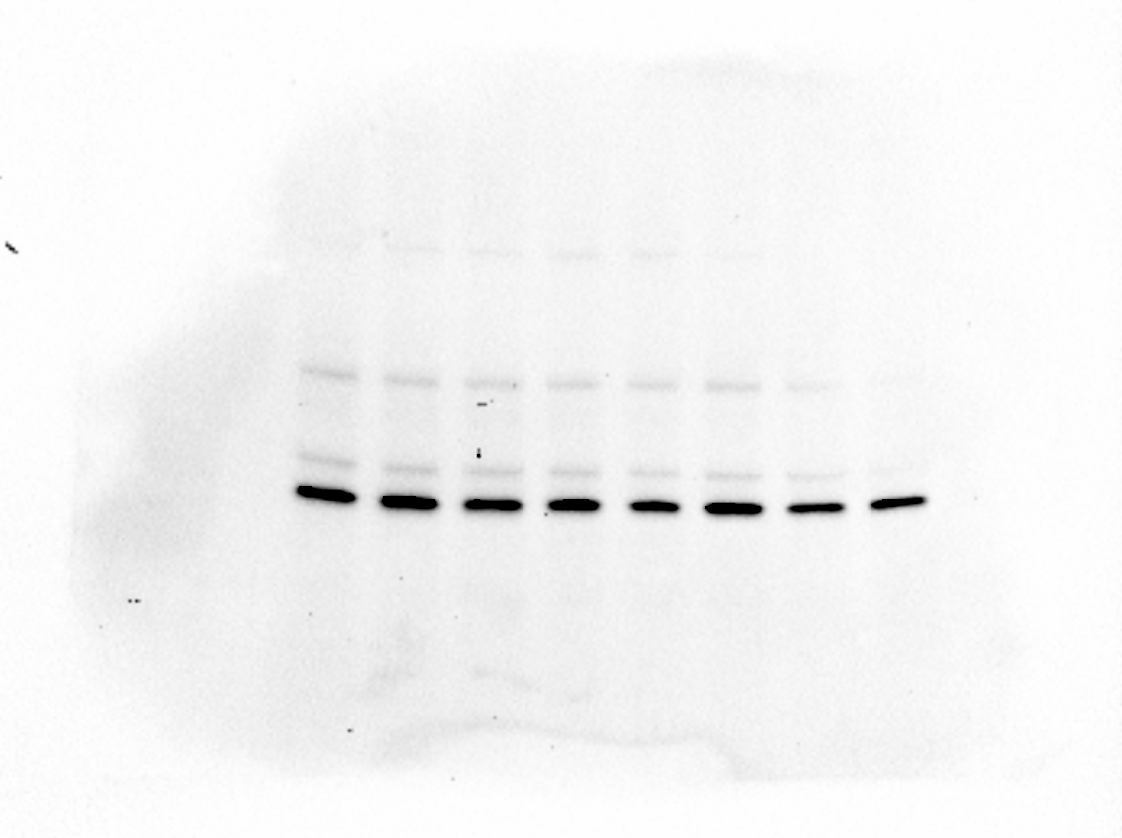

Supplement: Figure 4—source data 2. [file elife-90116-fig4-data2.zip › Fig.4 orignal images jpeg/2019-02-13 GAPDH.jpg]

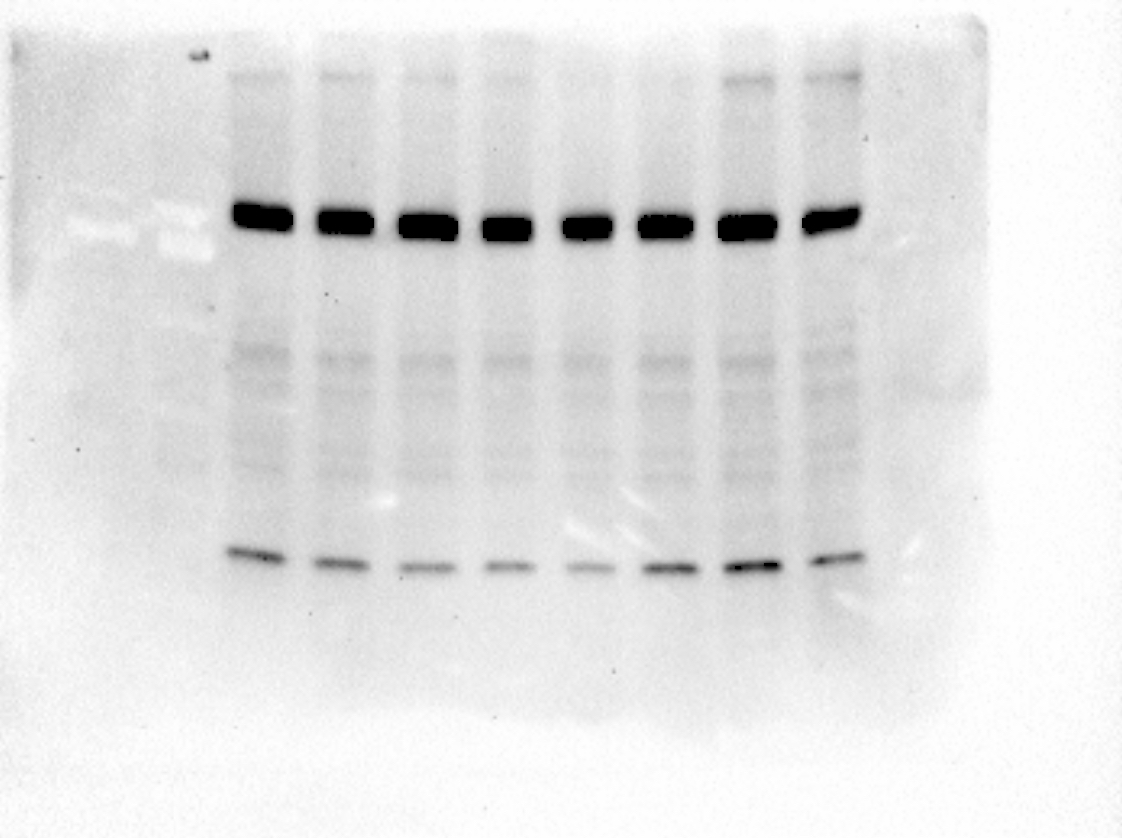

Supplement: Figure 4—source data 2. [file elife-90116-fig4-data2.zip › Fig.4 orignal images jpeg/2019-02-08 GluA2.jpg]

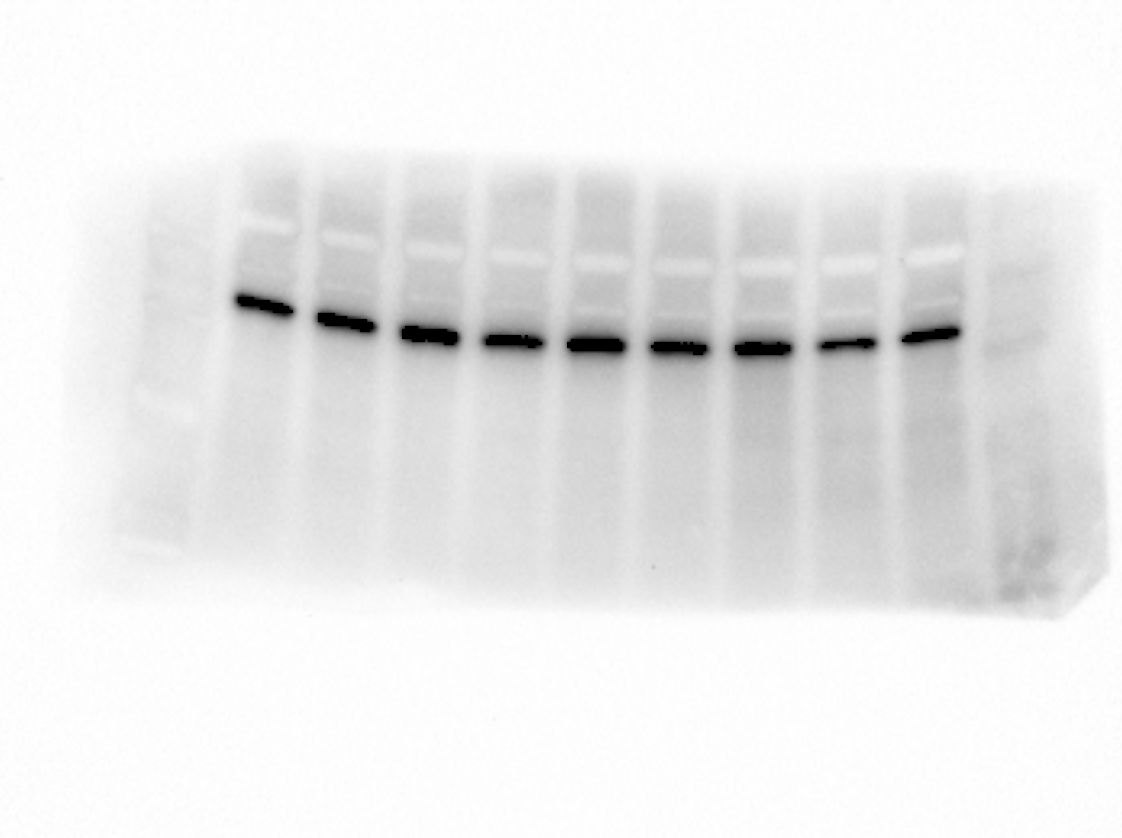

Supplement: Figure 4—source data 2. [file elife-90116-fig4-data2.zip › Fig.4 orignal images jpeg/2018-10-16_GAPDH.jpg]

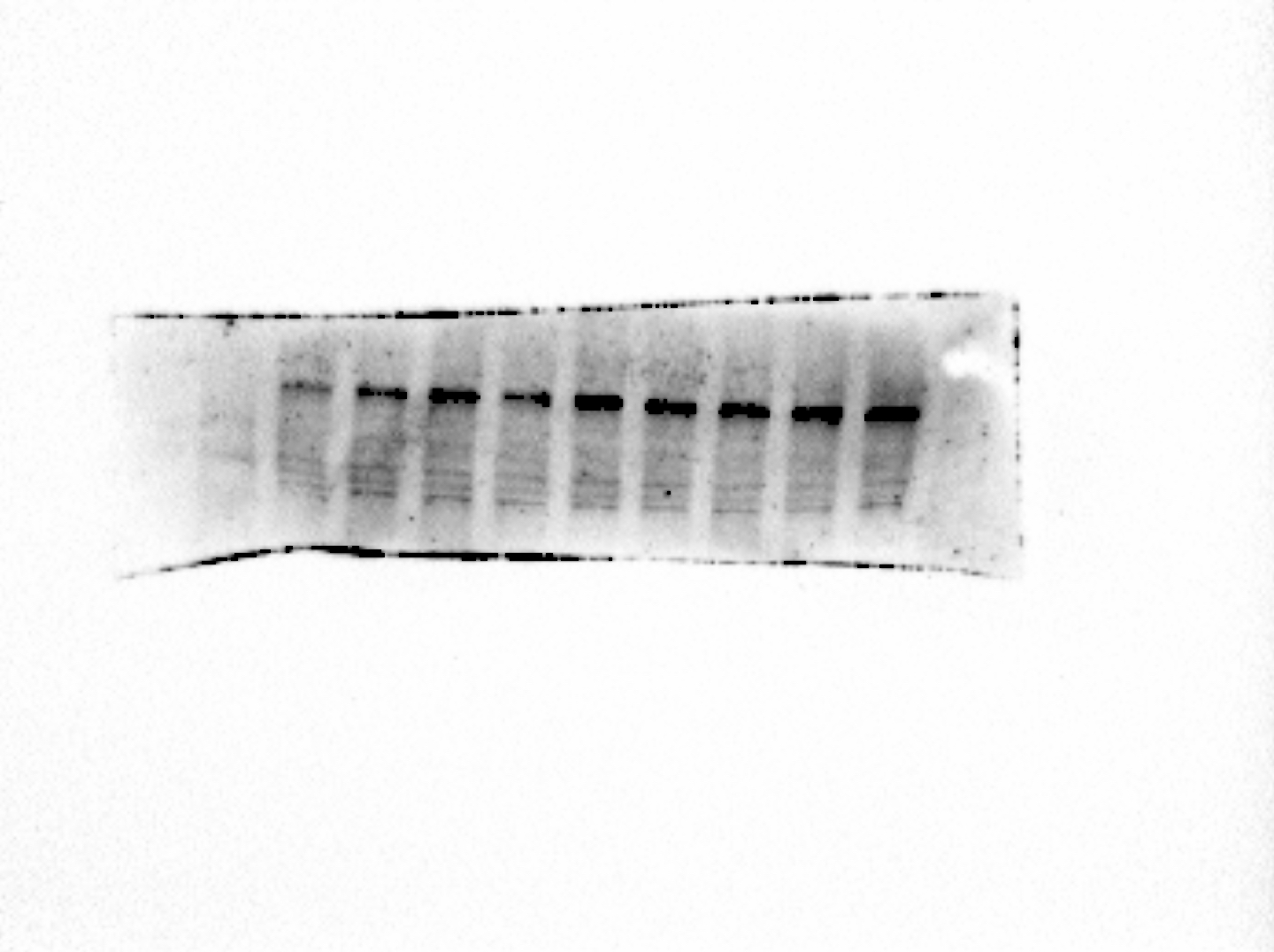

Supplement: Figure 4—source data 2. [file elife-90116-fig4-data2.zip › Fig.4 orignal images jpeg/2019-03-10 NR2B.jpg]

**Figure 5**

**G**

**CPEB1**

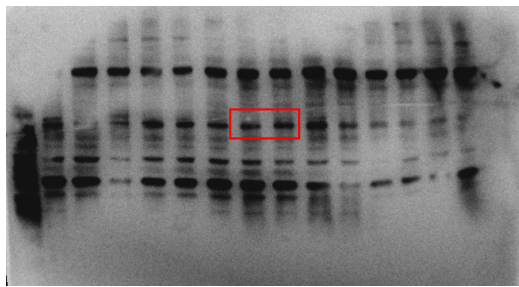

**CPEB4**

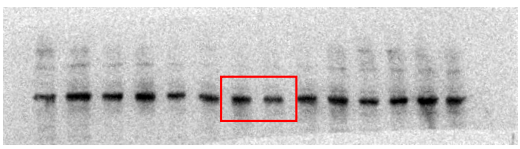

**GAPDH**

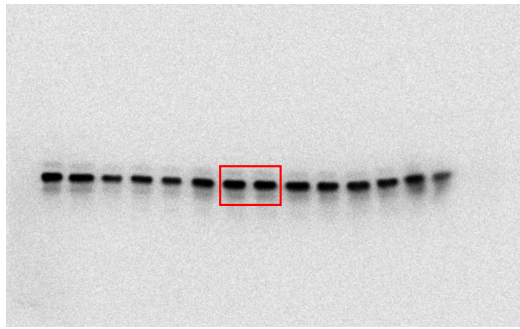

**I**

**CPEB3**

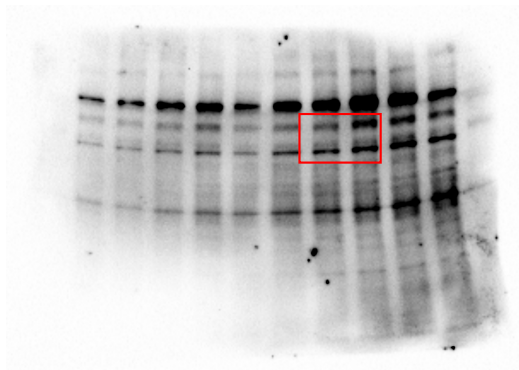

**GAPDH**

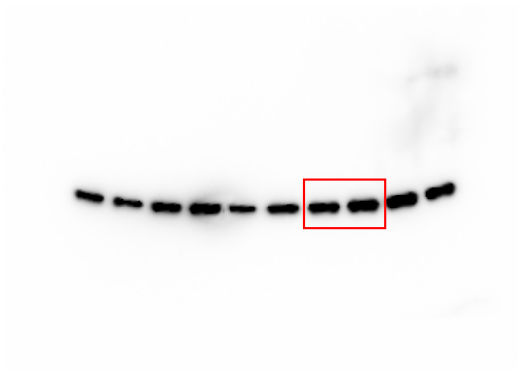

**L**

**GluA1**

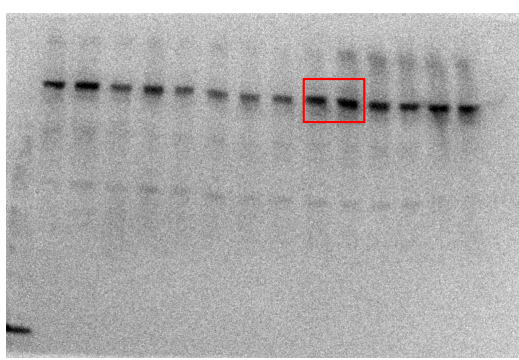

**GluA2**

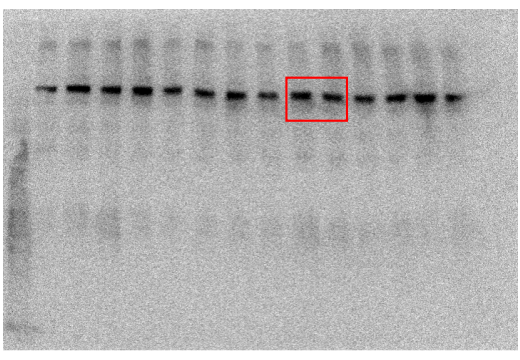

**PSD-95**

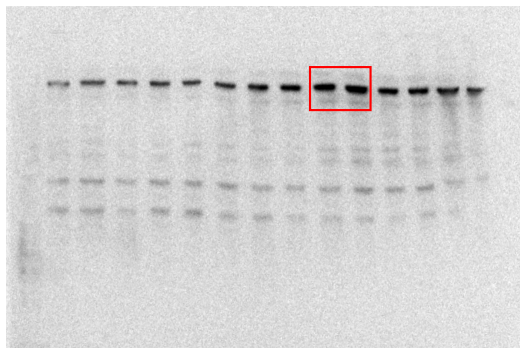

**NR2B**

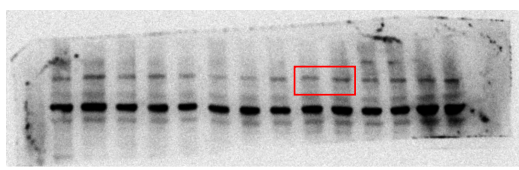

**GAPDH**

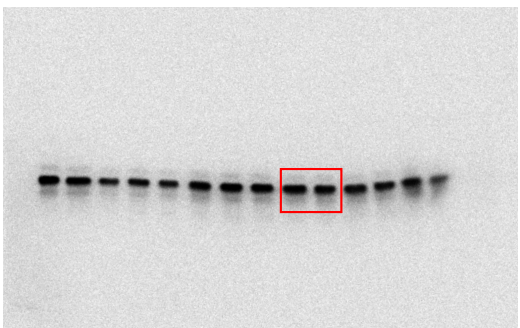

Supplement: Figure 5—source data 1. [file elife-90116-fig5-data1.zip › Fig. 5 source data/Fig.5 source data-2.pdf]

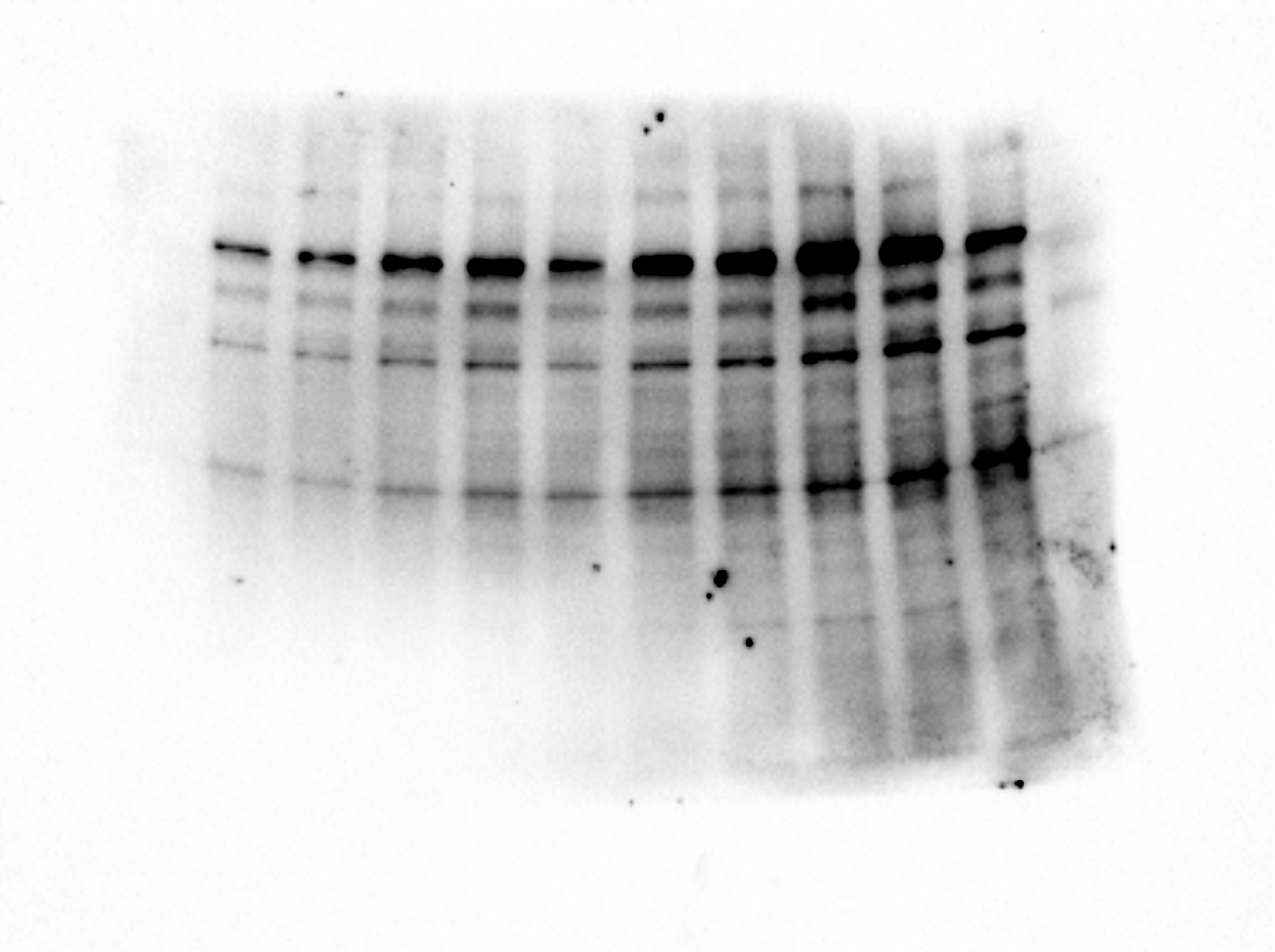

Supplement: Figure 5—source data 2. [file elife-90116-fig5-data2.zip › Fig.5 original images jpeg/2018-09-13 CPEB3.jpg]

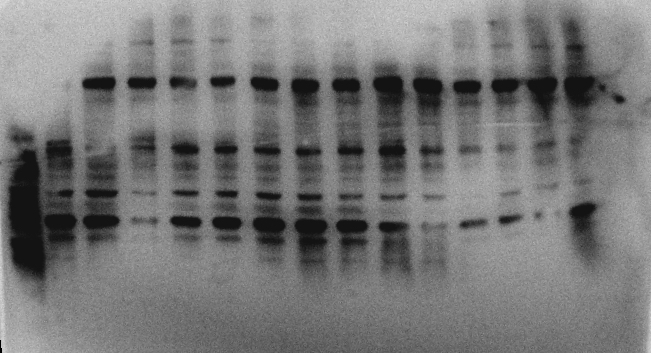

Supplement: Figure 5—source data 2. [file elife-90116-fig5-data2.zip › Fig.5 original images jpeg/2022-01-30 CPEB1.jpg]

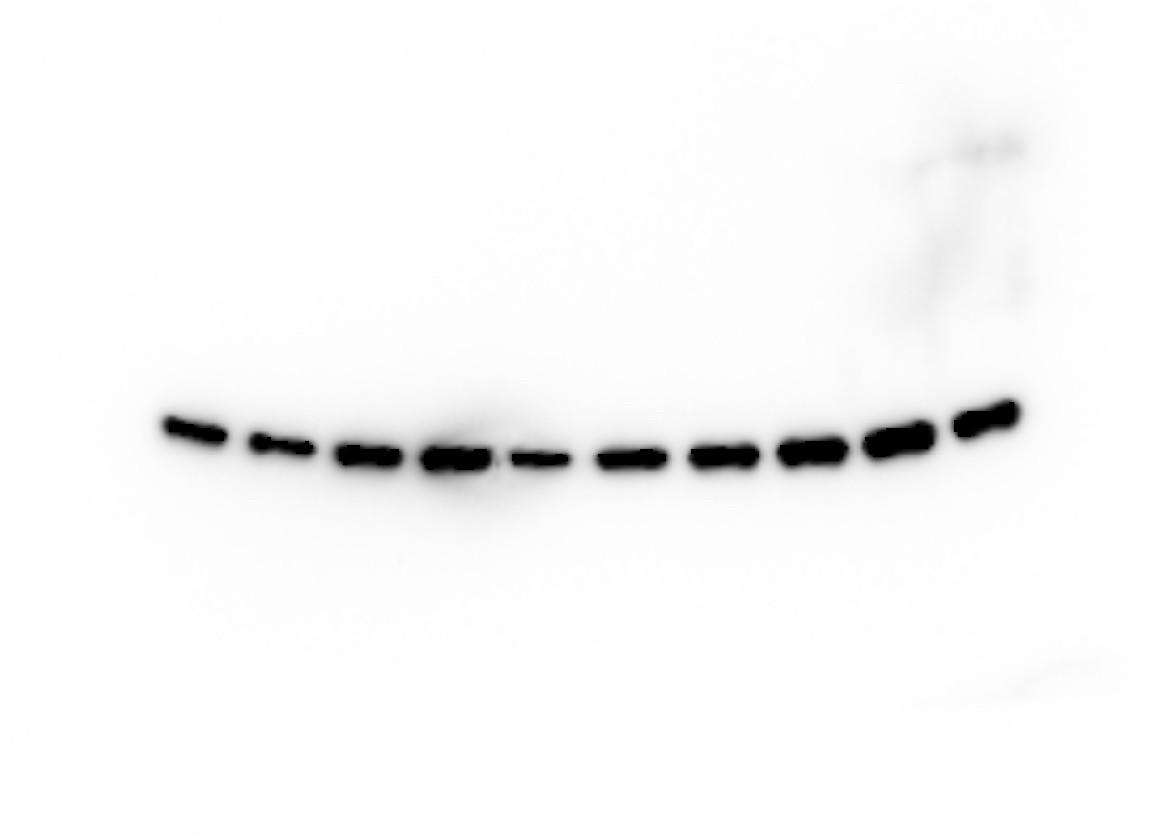

Supplement: Figure 5—source data 2. [file elife-90116-fig5-data2.zip › Fig.5 original images jpeg/2018-09-20 GAPDH.jpg]

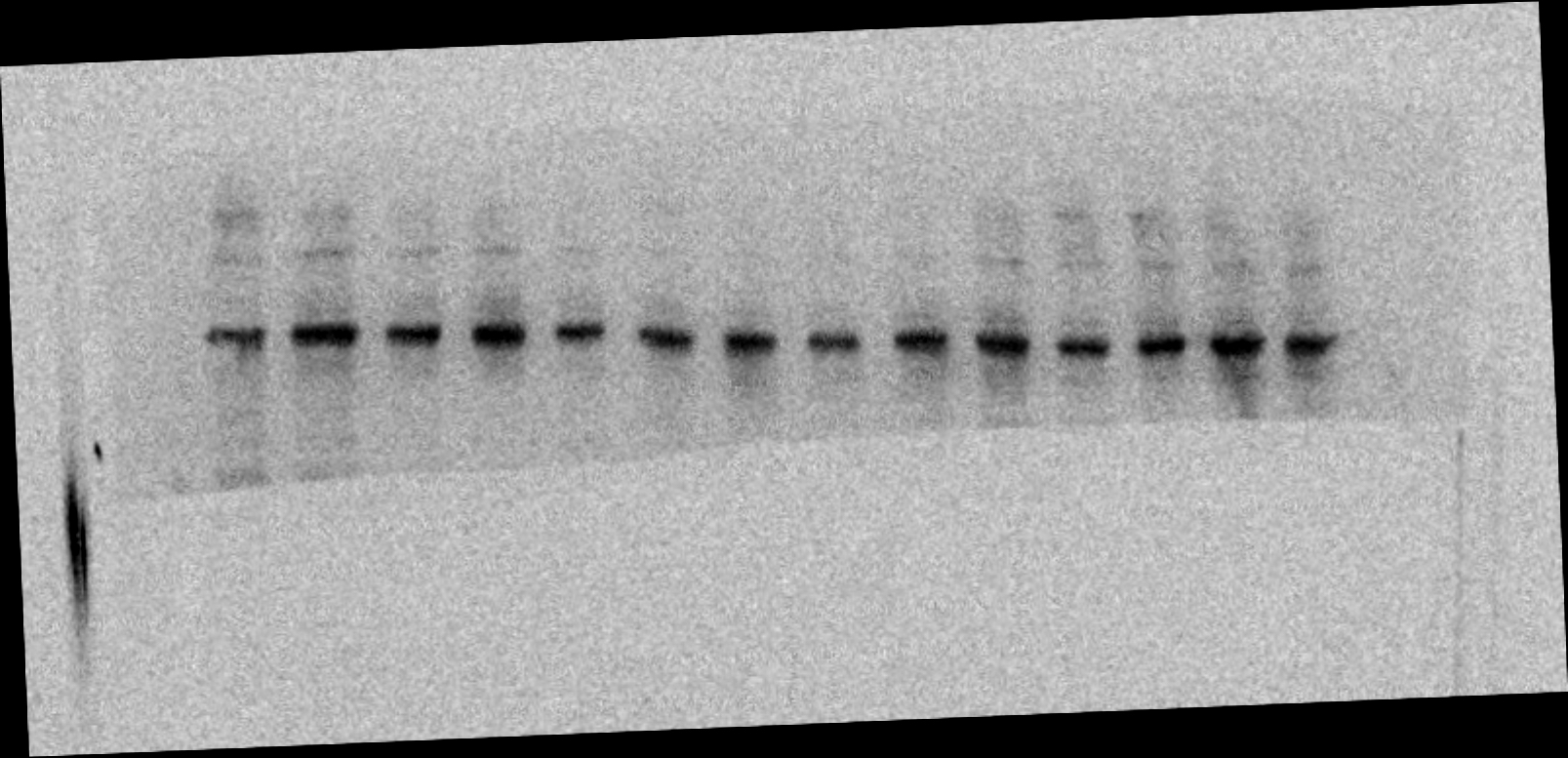

Supplement: Figure 5—source data 2. [file elife-90116-fig5-data2.zip › Fig.5 original images jpeg/2022-01-28 CPEB4.jpg]

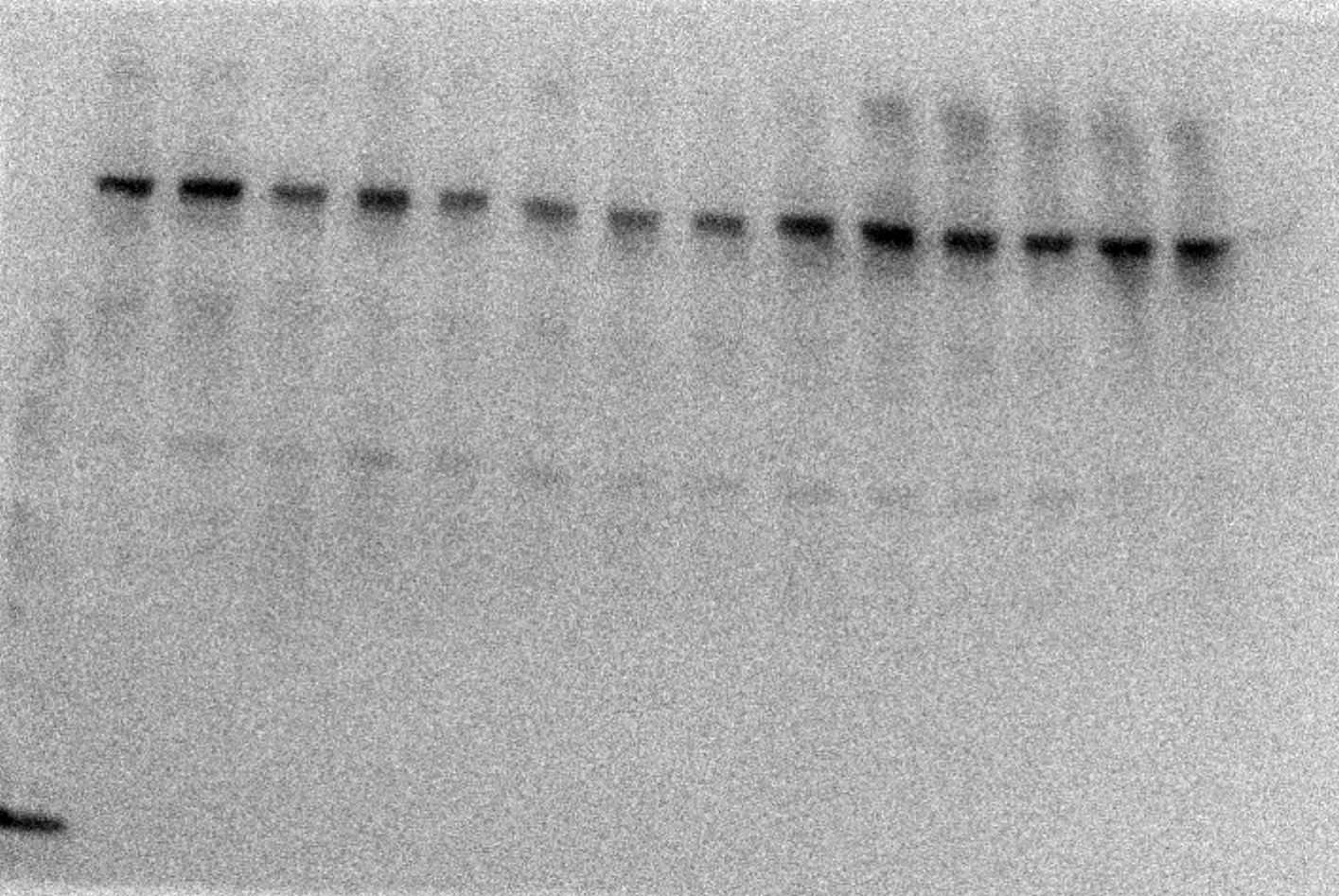

Supplement: Figure 5—source data 2. [file elife-90116-fig5-data2.zip › Fig.5 original images jpeg/2021-12-22 GluA1.jpg]

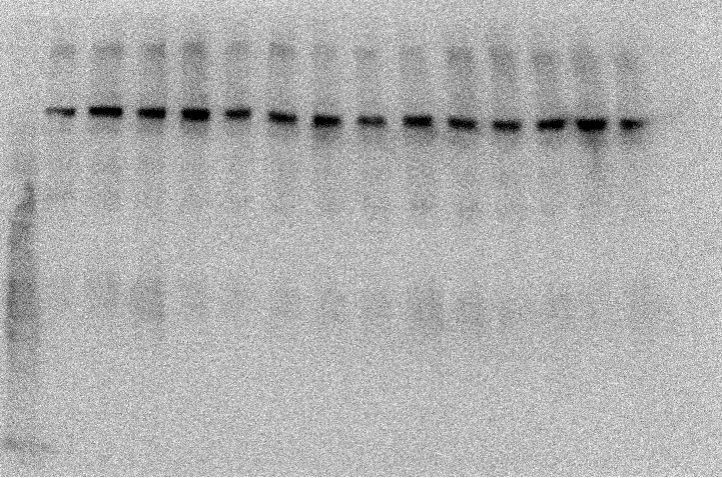

Supplement: Figure 5—source data 2. [file elife-90116-fig5-data2.zip › Fig.5 original images jpeg/2021-12-21 GluA2.jpg]

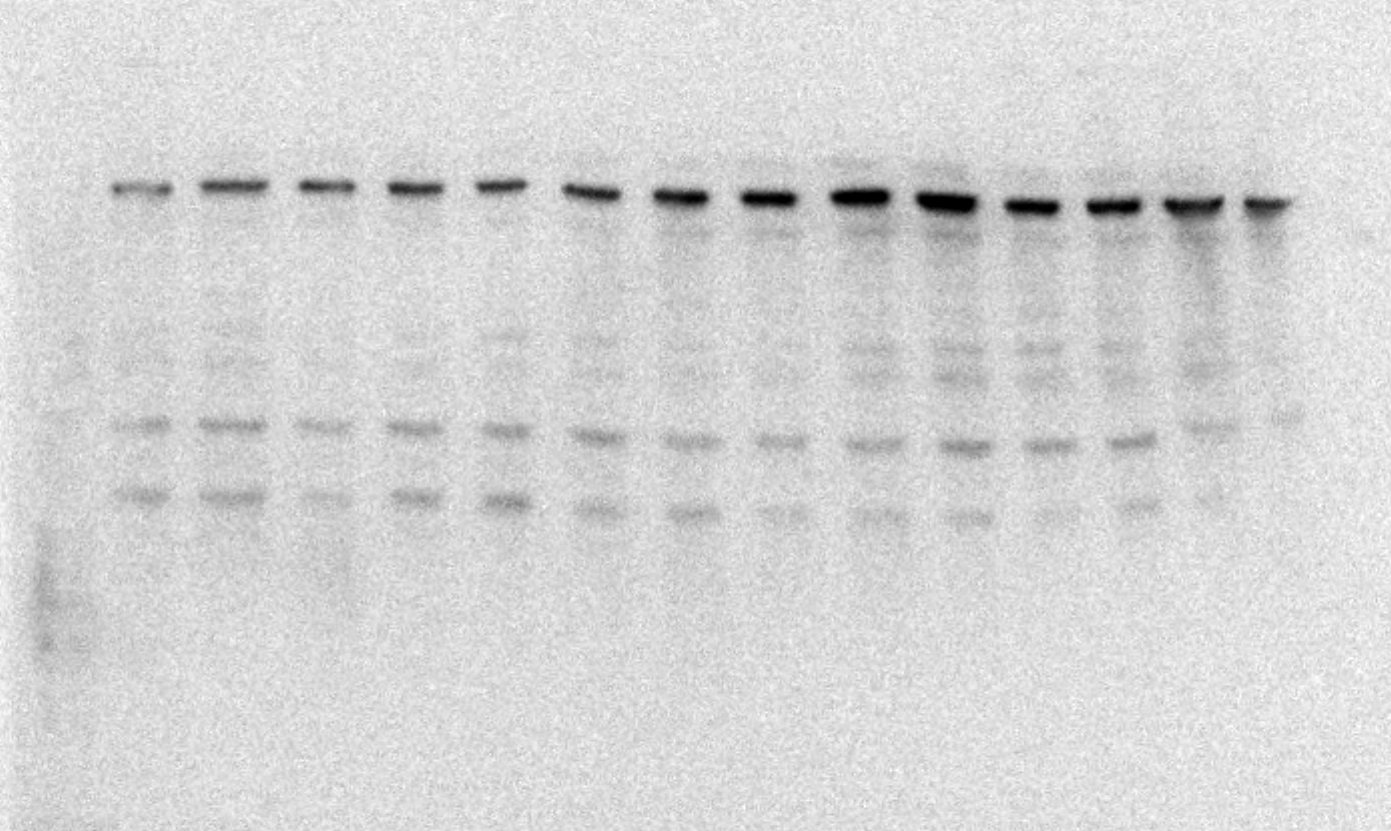

Supplement: Figure 5—source data 2. [file elife-90116-fig5-data2.zip › Fig.5 original images jpeg/2021-12-23 PSD95.jpg]

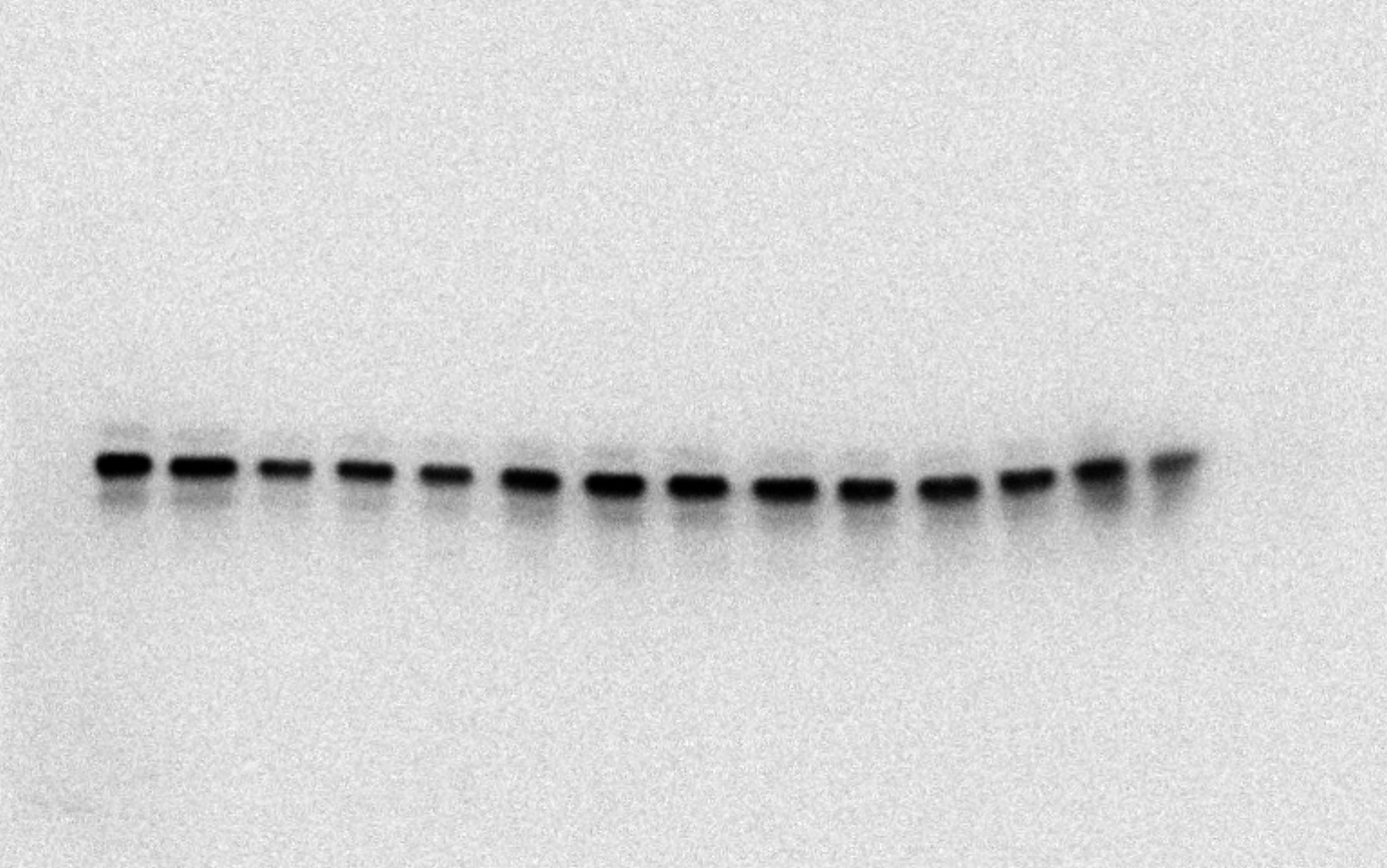

Supplement: Figure 5—source data 2. [file elife-90116-fig5-data2.zip › Fig.5 original images jpeg/2021-12-24 GAPDH.jpg]

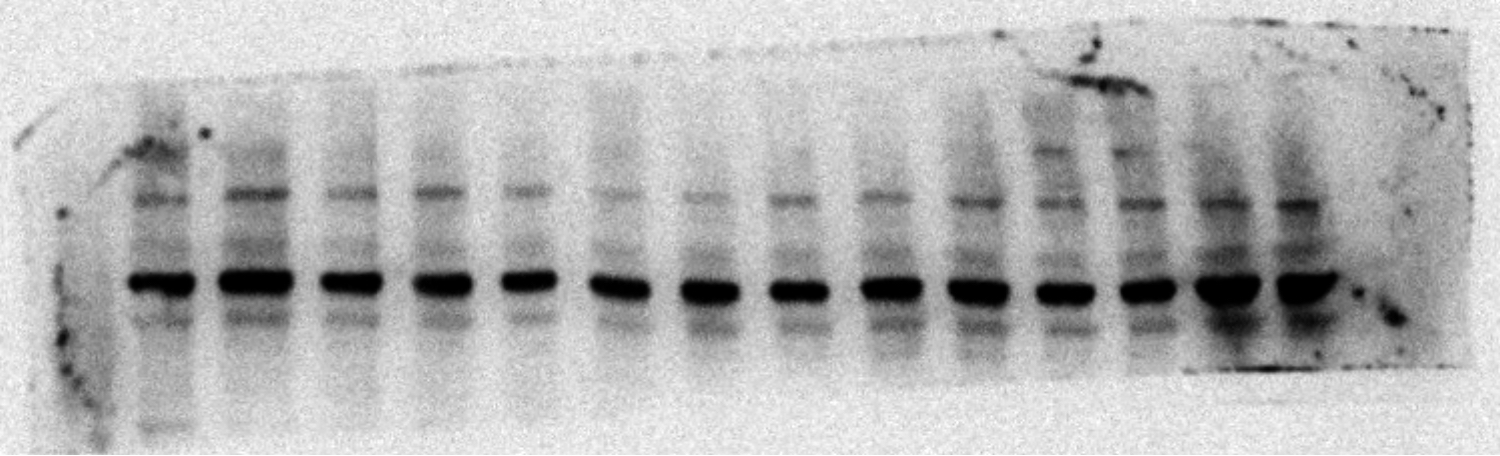

Supplement: Figure 5—source data 2. [file elife-90116-fig5-data2.zip › Fig.5 original images jpeg/2021-12-29 NR2B.jpg]

**Figure 7**

**A**

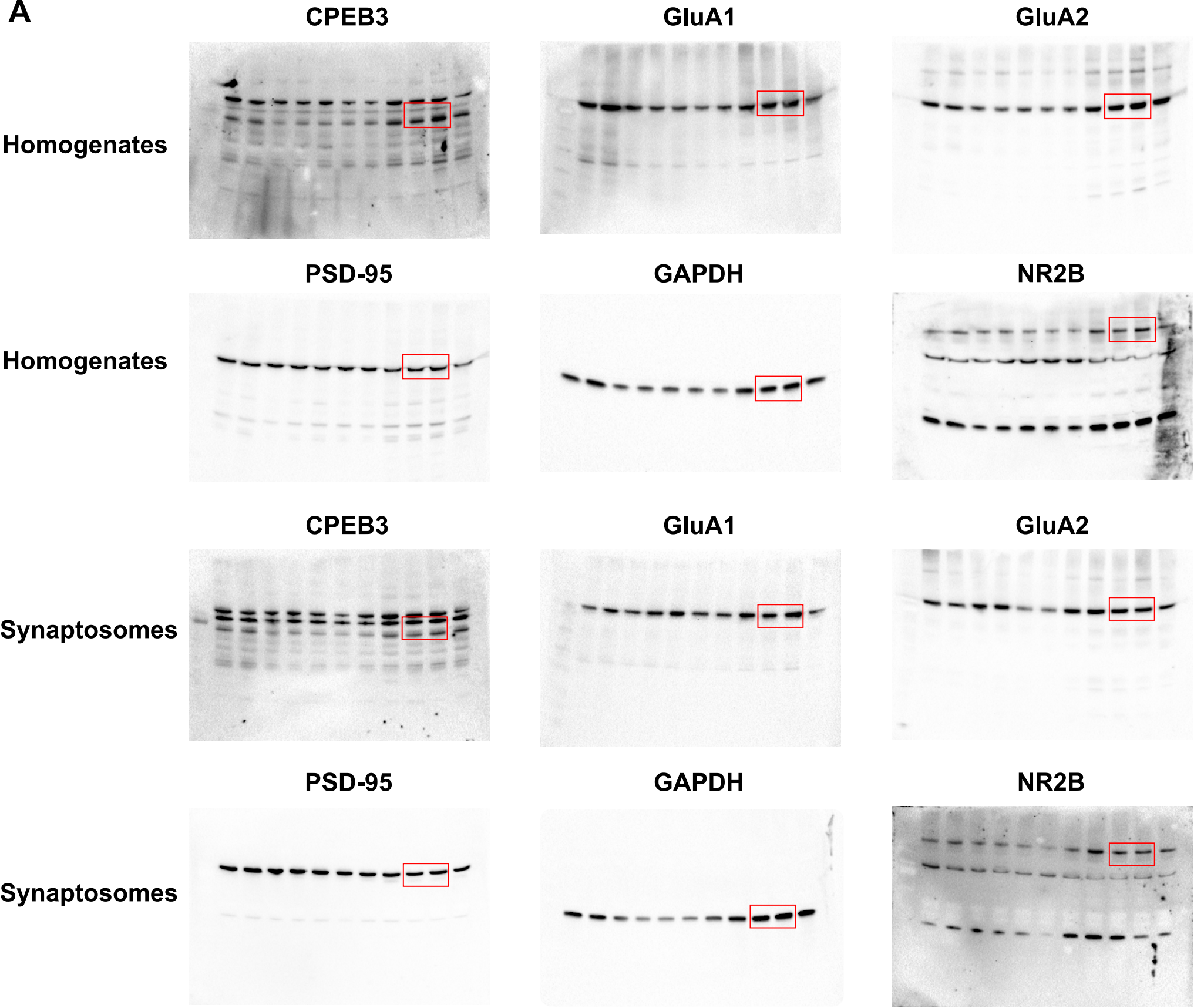

Supplement: Figure 7—source data 1. [file elife-90116-fig7-data1.zip › Fig. 7 source data/Fig.7 source data-2.pdf]

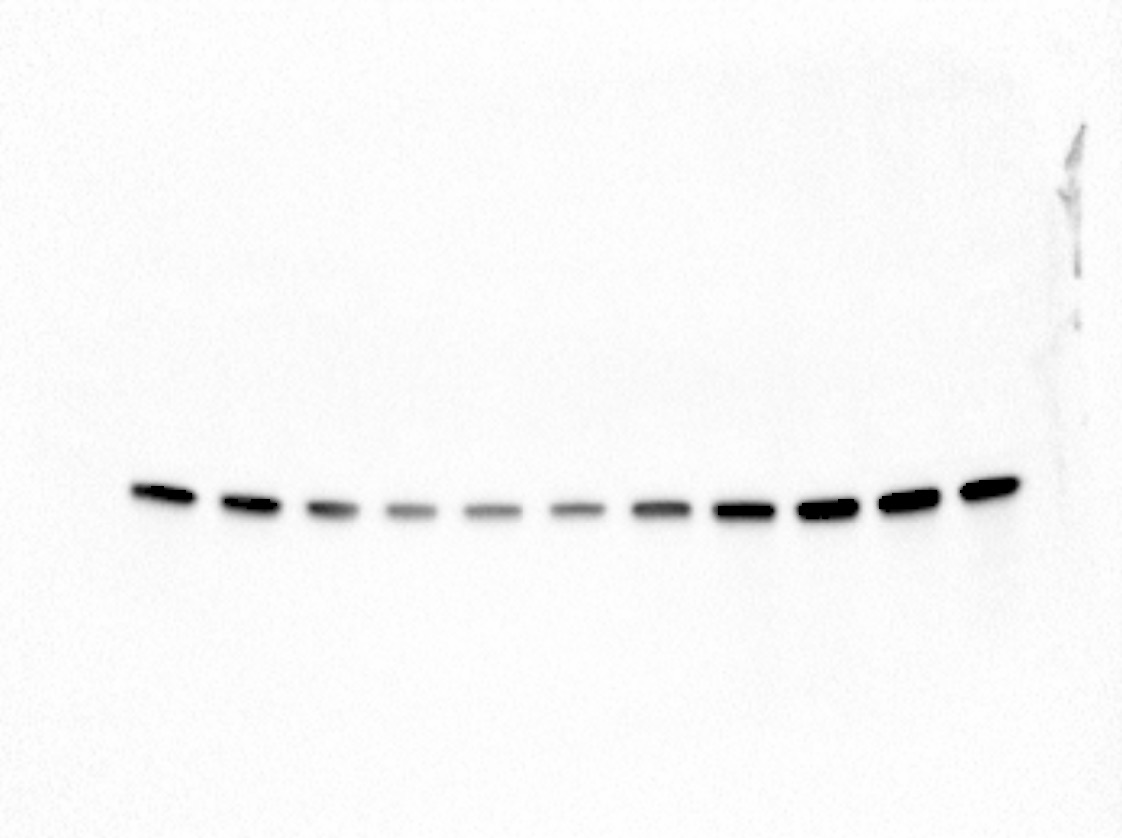

Supplement: Figure 7—source data 2. [file elife-90116-fig7-data2.zip › Fig.7 original images jpeg/GAPDH Sp 2019-08-23.jpg]

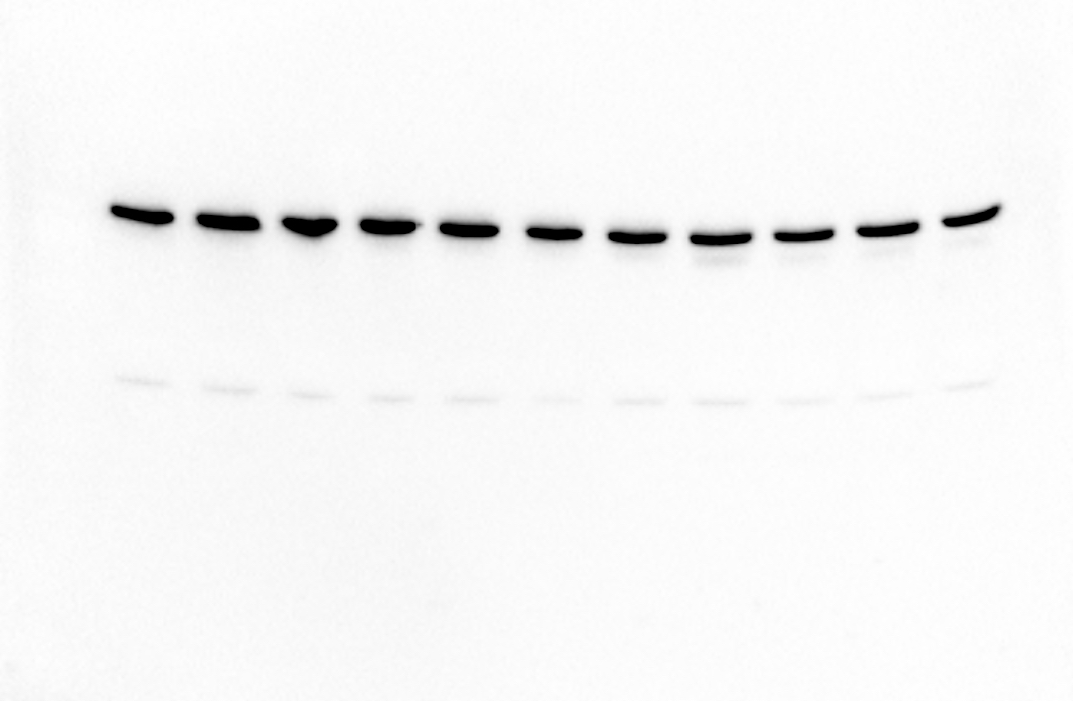

Supplement: Figure 7—source data 2. [file elife-90116-fig7-data2.zip › Fig.7 original images jpeg/PSD95 Sp 2019-08-19.jpg]

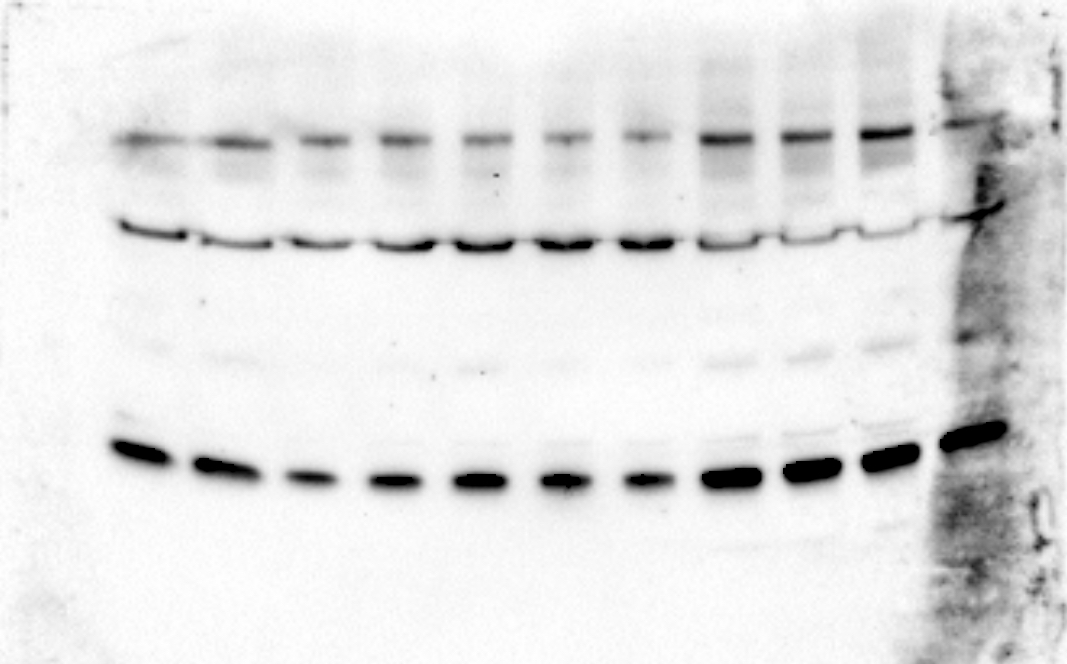

Supplement: Figure 7—source data 2. [file elife-90116-fig7-data2.zip › Fig.7 original images jpeg/NR2B Hp 2019-08-12.jpg]

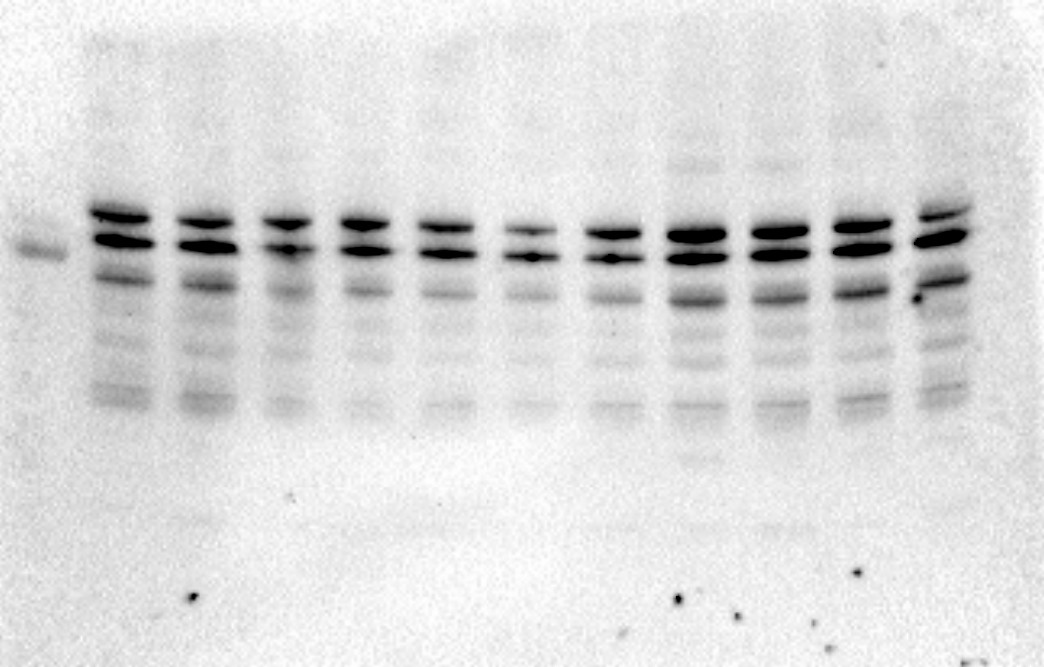

Supplement: Figure 7—source data 2. [file elife-90116-fig7-data2.zip › Fig.7 original images jpeg/CPEB3 Sp 2019-08-18.jpg]

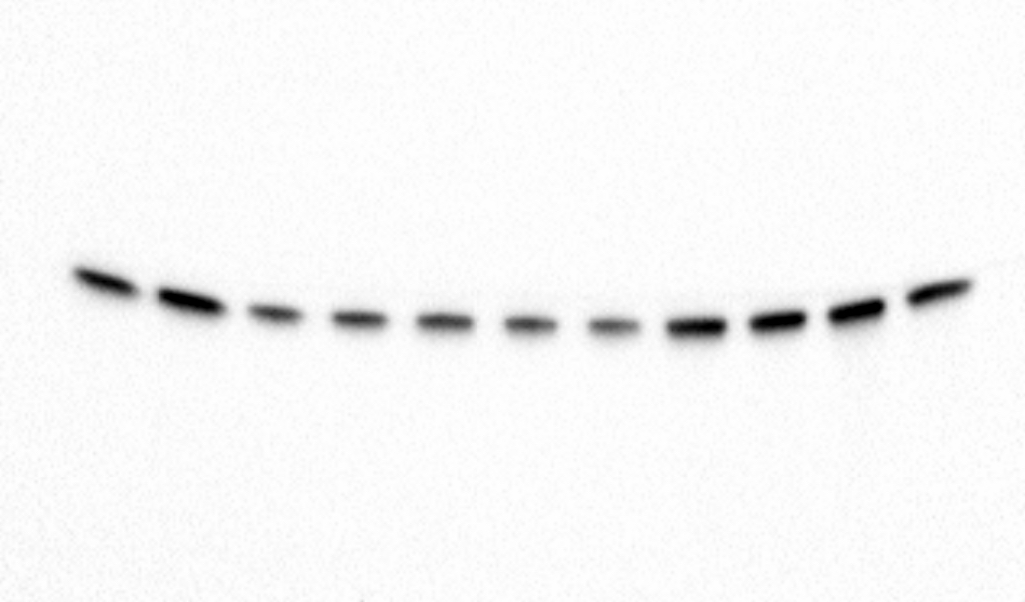

Supplement: Figure 7—source data 2. [file elife-90116-fig7-data2.zip › Fig.7 original images jpeg/GAPDH Hp 2019-08-11.jpg]

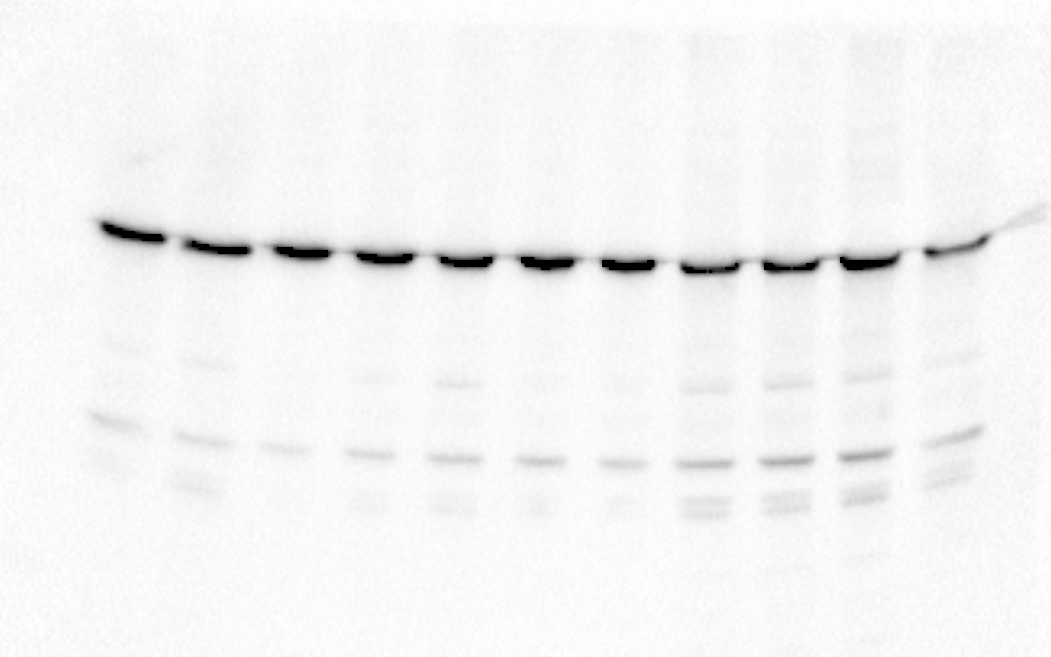

Supplement: Figure 7—source data 2. [file elife-90116-fig7-data2.zip › Fig.7 original images jpeg/PSD95 Hp 2019-08-10.jpg]

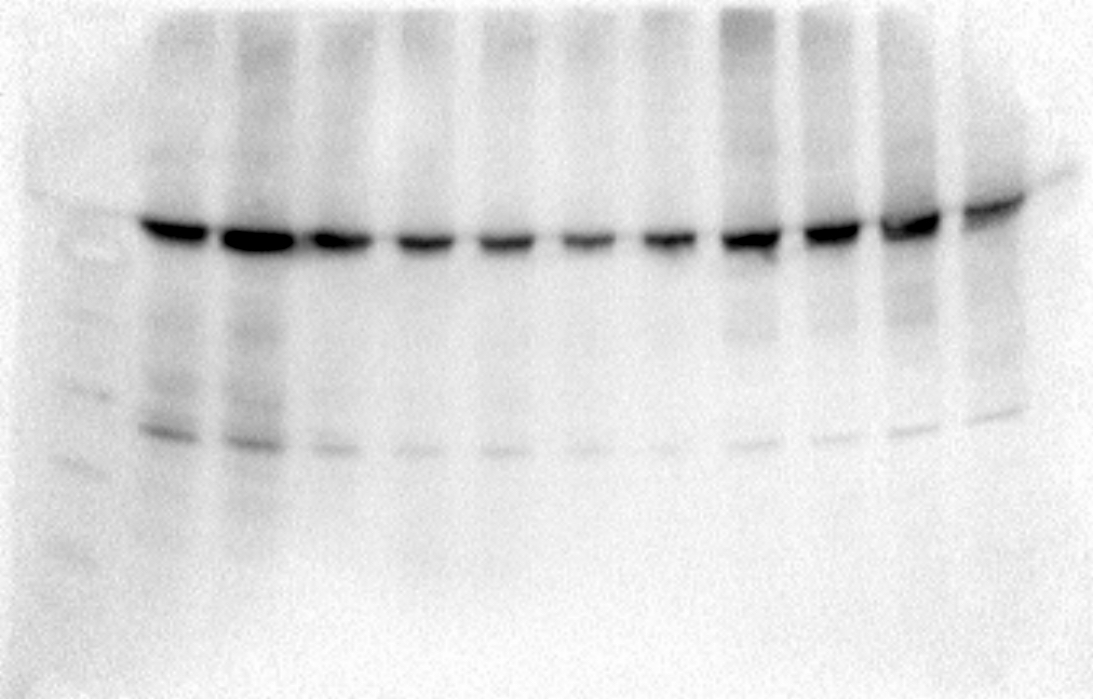

Supplement: Figure 7—source data 2. [file elife-90116-fig7-data2.zip › Fig.7 original images jpeg/GluA1 Hp 2019-08-09.jpg]

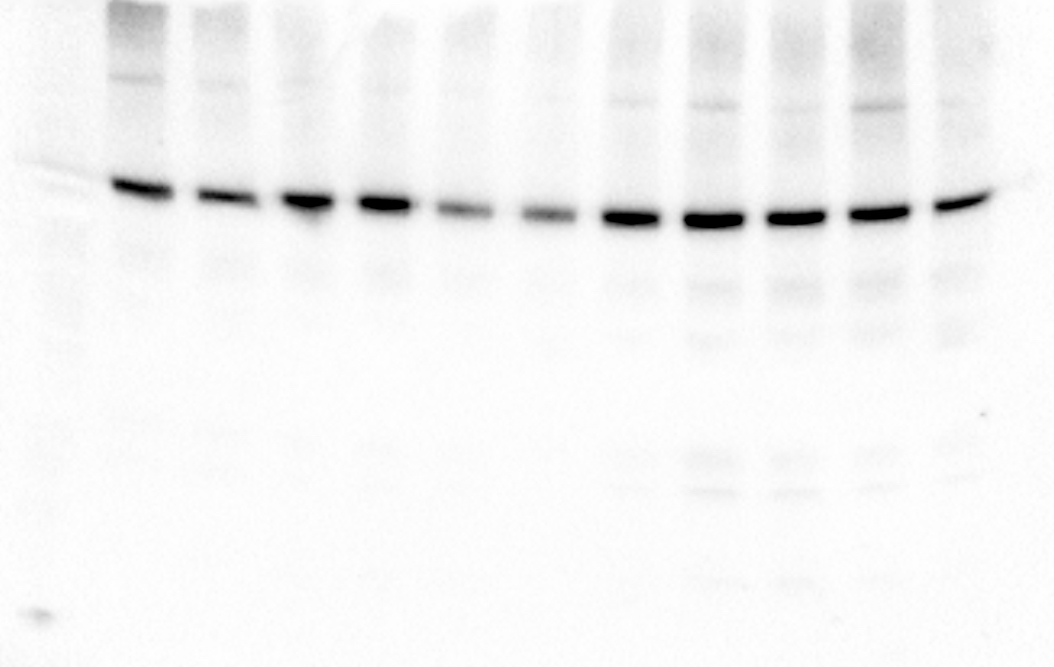

Supplement: Figure 7—source data 2. [file elife-90116-fig7-data2.zip › Fig.7 original images jpeg/GluA2 Sp 2019-08-20.jpg]

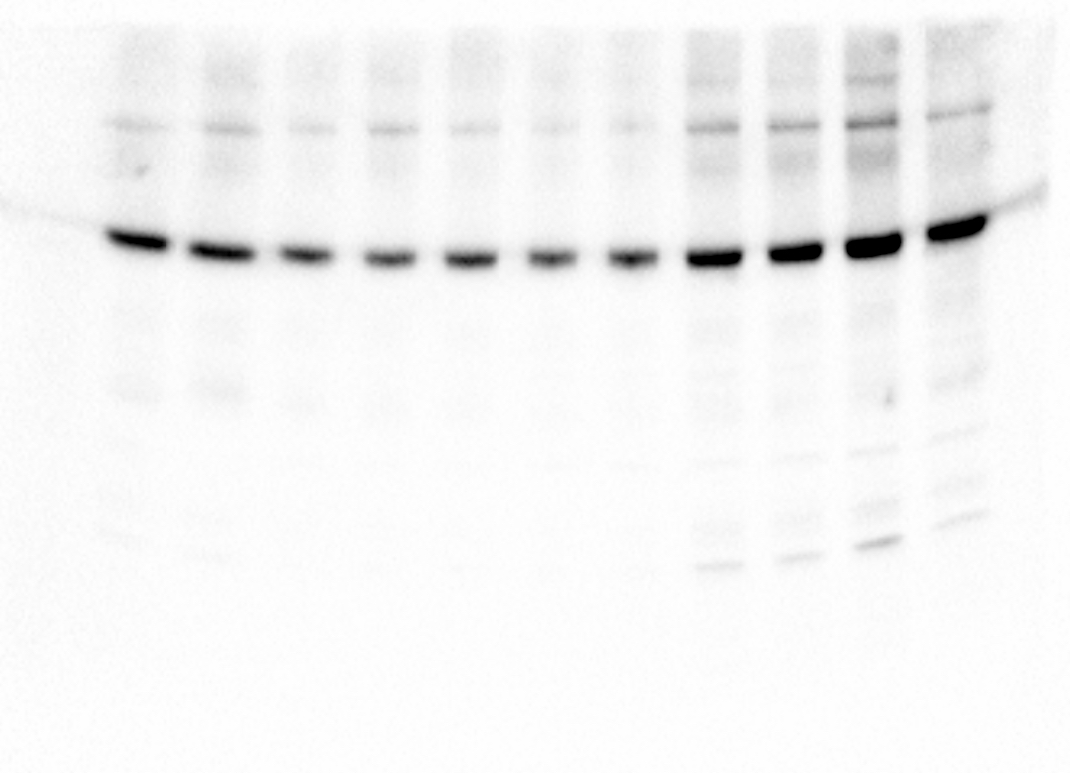

Supplement: Figure 7—source data 2. [file elife-90116-fig7-data2.zip › Fig.7 original images jpeg/Differential uptake, kinetics and mechanisms of intracellular trafficking of next-generation antisense oligonucleotides across human cancer cell lines SI.jpg]

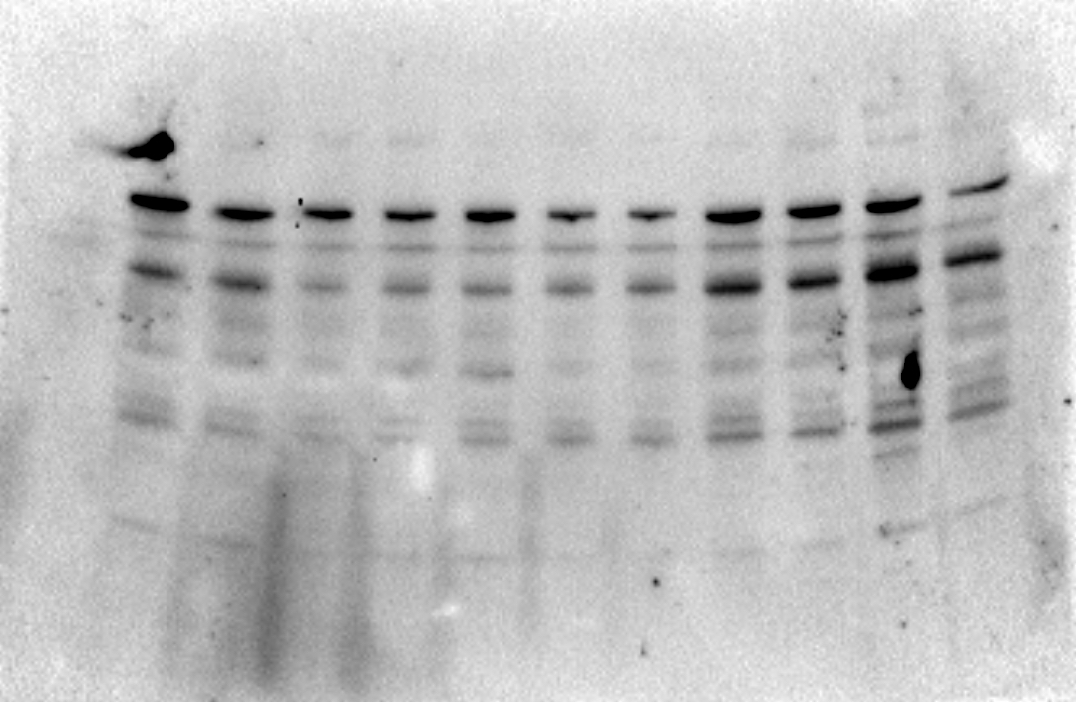

Supplement: Figure 7—source data 2. [file elife-90116-fig7-data2.zip › Fig.7 original images jpeg/CPEB3 Hp 2019-08-07.jpg]

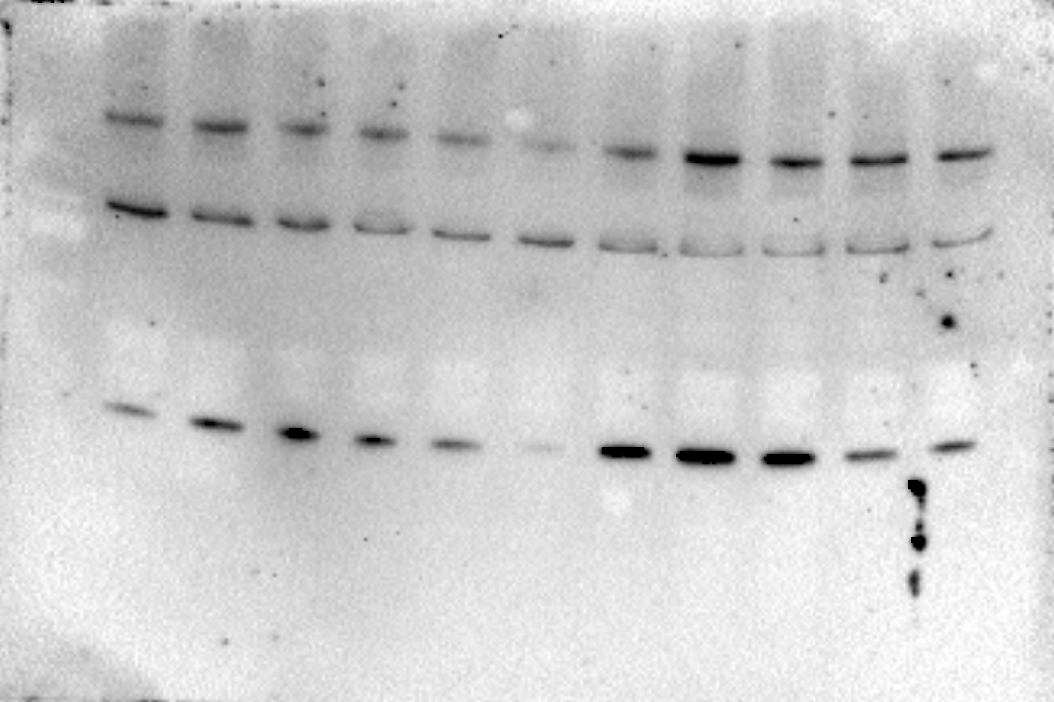

Supplement: Figure 7—source data 2. [file elife-90116-fig7-data2.zip › Fig.7 original images jpeg/NR2B Sp 2019-08-22.jpg]

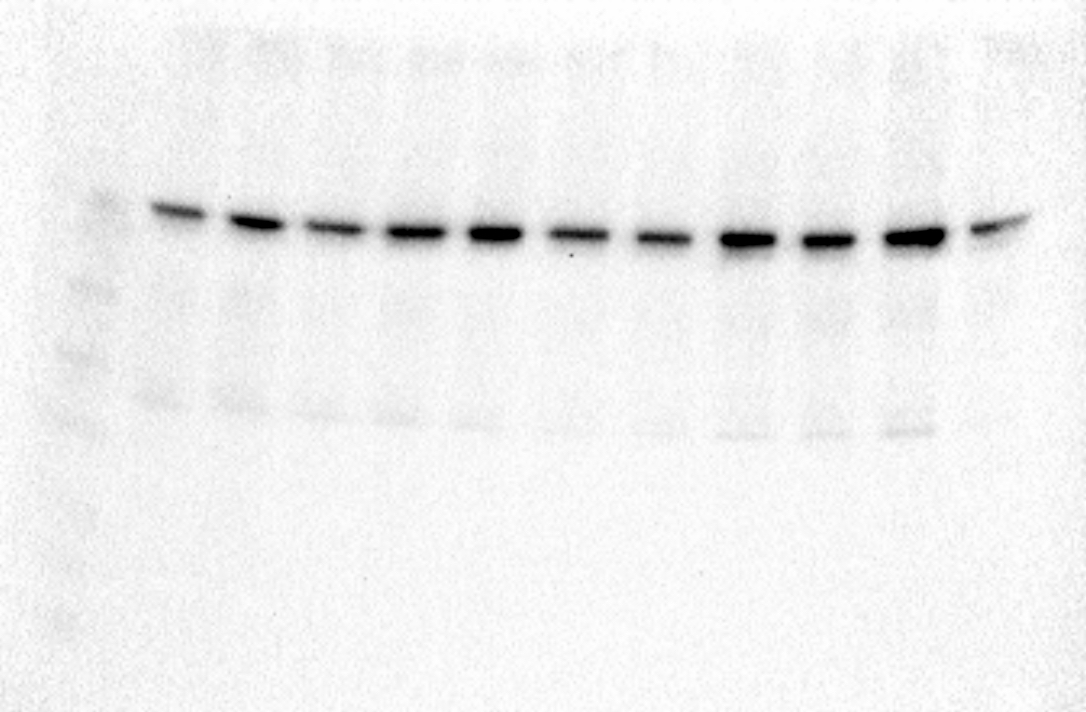

Supplement: Figure 7—source data 2. [file elife-90116-fig7-data2.zip › Fig.7 original images jpeg/GluA1 Sp 2019-08-21.jpg]
